# Proceedings of Seventh Annual Session of I. H. A.

**Published:** 1887-06

**Authors:** 


### BOOK NOTICES.

### Proceedings of the Seventh Annual Session of the International Hahnemannian Association.

"Better late than never" is an old saying, and one we presume may be applied to the tardy appearance of this volume! The report here given of the last meeting of the I. H. A. is fairly complete, and is well arranged.

In variety and value the papers are unusually good, and the volume will amply repay careful study. Where all the papers are so well written and of such practical value, it would be impossible for us to notice any one in particular. All are good, each in its department.

We have received several complaints from members who took part in the Saratoga meeting that the reports have been "doctored" by some one. This is a serious matter, for the papers and the discussions should be honestly reported and printed. We would suggest that the Association should see that this is done in future, and that the Secretary have charge of the printing.

